# IL-7 abrogates memory T regulatory cell functions by modulation of CD39/ATP axis in vitro and in vivo in HIV infected and non-infected patients

**DOI:** 10.1186/1742-4690-9-S2-P290

**Published:** 2012-09-13

**Authors:** M Younas, S Hue, M Surenaud, C Lacabaratz, A Guiguin, A Wiedman, S Becq, T Croughs, J Lelievre, Y Levy

**Affiliations:** 1 Faculté de médecine, Université Paris-Est, Créteil, Créteil, France; 2 Cytheris, France

## Background

IL-7 cytokine regulate the expansion and maturation of T cells. The good safety profile of IL-7 raises the opportunity of its use as a vaccine adjuvant. However, its role on the regulation of T cell responses has not been explored. We investigated the effects of IL-7 on regulatory T cell (Treg) functions invitro and invivo.

## Methods

Treg and CD8+ T cells were isolated from healthy donors (n=10) (HD) and chronically HAART treated HIV+ pt enrolled in a phase I/II IL-7 INSPIRE study (n=6X) (Levy et al, CID, in press). Treg subpopulations (naïve CD45RA+FoxP3++CD45RA+CD25++CD127+/-, memory (mTreg) Foxp3highCD45RA-CD25highCD127low, FoxP3++CD45RA-CD25++CD127+/-) were cultured with IL-7 (10 ng/ml). Phenotype (CD39, Bcl-2, Stat5P, purinergic receptor P2X7R), suppression of the proliferation of autologous antiCD3 activated CD8+ T cells (CFSE stained) and cytokine profile (IL-17 production) of Treg were analyzed.

## Results

In HD, IL-7 induces expression of STAT5 and BCL-2 on all Treg populations. IL-7 reduces the suppressive effects of mTreg on CD8+ proliferation (% CFSE low w/wo IL7 was 15% and 40%, respectively, n=4, P=0.01). This effect was associated with a down-modulation of CD39 enzyme (MFI 65 vs 90 w/wo IL7, P=0.01) and an increase of P2X7R expression. IL-7 effect was reproduced using anti-CD39 blocking antibody and PPAD (inhibitor of P2X7R). IL-7 incubated Treg switched to a Th17 phenotype as assessed by the increase of Th17 production and RORgC expression. IL-7 treated patients exhibited a decrease of the frequency of Treg/CD39+ and an increase of RORgC mRNA in PBMCs as compared to pre-IL-7 therapy.

## Conclusion

IL-7 relieves the suppressive effect of mTreg through a modulation of the CD39/ATP axis. By increasing P2X7R expression, IL-7 increases the susceptibility of these cells to ATP, a trigger of Th17 differentiation. An effect also observed in IL-7 treated patients. These results suggest that IL-7 could be used as adjuvant to reinforce T cell responses.

